# Impaired implantation as a major upstream pathway of preeclampsia: a narrative synthesis of mechanistic, epidemiological and biomarker evidence

**DOI:** 10.3389/frph.2025.1743504

**Published:** 2026-01-05

**Authors:** Abubakar Ibrahim, Engku Husna Engku Ismail, Martina Irwan Khoo, Lukman Yusuf, Nik Hazlina Nik Hussain, Anani Aila Mat Zin, Liza Noordin, Sarimah Abdullah, Zaleha Abdullah Mahdy, Nik Ahmad Zuky Nik Lah

**Affiliations:** 1 Department of Obstetrics and Gynaecology, School of Medical Sciences, Universiti Sains Malaysia, Kubang Kerian, Kelantan, Malaysia; 2 Department of Chemical Pathology, School of Medical Sciences, Universiti Sains Malaysia, Kubang Kerian, Kelantan, Malaysia; 3 Department of Pathology, School of Medical Sciences, Universiti Sains Malaysia, Kubang Kerian, Kelantan, Malaysia; 4 Women’s Health Development Unit, School of Medical Sciences, Universiti Sains Malaysia, Kubang Kerian, Kelantan, Malaysia; 5 Department of Physiology, School of Medical Sciences, Universiti Sains Malaysia, Kubang Kerian, Kelantan, Malaysia; 6 Biostatistics and Research Methodology Unit, School of Medical Sciences, Universiti Sains Malaysia, Kubang Kerian, Kelantan, Malaysia; 7 Department of Obstetrics and Gynaecology, Hospital Canselor Tuanku Muhriz UKM, Jalan Yaacob Latif Kuala Lumpur, Cheras, Wilayah Persekutuan Kuala Lumpur, Malaysia

**Keywords:** corpus luteum, decidualization resistance, frozen embryo transfer, immune tolerance, implantation failure, placental biomarkers, preeclampsia PE, seminal plasma

## Abstract

Preeclampsia (PE) remains a major cause of maternal and perinatal morbidity worldwide. Although abnormal placentation and shallow trophoblast invasion are well recognized, increasing evidence suggests that the origins of PE lie earlier, at the stage of implantation and decidualization. A deeper understanding of impaired implantation as the initiating event offers new opportunities for prediction, prevention, and therapy. This narrative review synthesizes mechanistic, epidemiological, and biomarker evidence accumulated over the past two years. Mechanistic studies reveal that defective decidualization and resistance to progesterone signaling impair stromal cell differentiation, angiogenic balance, and vascular remodeling. Immunological dysregulation, including maladaptive KIR–HLA interactions, CD40–CD40L pathway activation, and altered cytokine tolerance, further disrupts maternal–fetal communication. Clinical epidemiology strongly implicates implantation context: programmed frozen embryo transfer cycles lacking a corpus luteum consistently increase the risk of hypertensive disorders, highlighting the importance of peri-conception physiology. First-trimester biomarkers such as low PAPP-A, reduced PlGF, and abnormal uterine artery Doppler indices capture the early “fingerprint” of impaired implantation long before clinical disease. Emerging evidence also supports seminal plasma as a key modulator of immune priming and endometrial receptivity, with reduced exposure linked to higher PE risk. Together, these findings reframe PE not solely as a disorder of placental development in mid-gestation but as a disease with origins in implantation biology. By bringing together molecular, immunological, and clinical evidence, this review positions impaired implantation as a central trigger of PE. Recognition of implantation-era events as the upstream pathway provides a new framework for translational research, emphasizing peri-conception exposures, assisted reproduction practices, and biomarker discovery. Clinically, it highlights novel opportunities for early risk stratification and prevention strategies. This implantation-centered model may help shift the paradigm of PE from late-pregnancy diagnosis toward early-pregnancy prediction and intervention.

## Graphical Abstract

**Figure:**
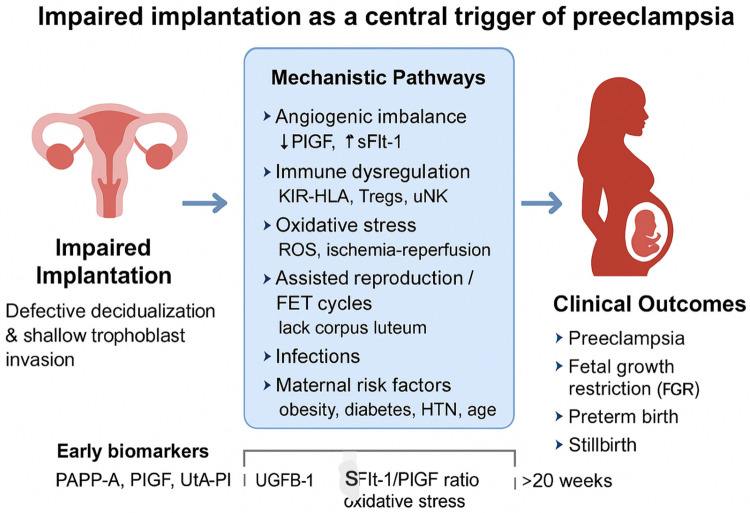

## Highlights

- Integrates ART/programmed-FET epidemiology (corpus luteum absence) with seminal-plasma exposure biology and immune receptor interactions.
- Brings recent multi-omic findings on decidualization resistance into a single mechanistic model connecting implantation-era events to PE phenotypes.
- Prioritizes concrete translational steps (preconception cohorts, peri-implantation biomarkers, trial-ready interventions) rather than only describing pathology.
- Proposes targeted mechanistic experiments (e.g., CD40–CD40L pathway interrogation in human decidua and seminal-plasma functional assays) to close evidence gaps.

## 1 Introduction

PE is a multifactorial pregnancy-specific disorder characterized by new-onset hypertension and proteinuria or end-organ dysfunction after the 20th week of gestation (1). Globally, PE affects approximately 2%–8% of pregnancies and is a leading cause of maternal and perinatal morbidity and mortality (2). According to the World Health Organization (WHO), PE and related hypertensive disorders account for an estimated 46,000 maternal deaths and 500,000 fetal or newborn deaths annually (2). In regions such as Asia and Africa, these conditions are responsible for about 10% of maternal deaths, with the proportion rising to approximately 25% in certain areas (2). The International Federation of Gynecology and Obstetrics (FIGO) recommends classifying PE based on gestational age at delivery, distinguishing between early-onset PE (delivery before 34 weeks), preterm PE (delivery before 37 weeks), late-onset PE (delivery at or after 34 weeks), and term PE (delivery at or after 37 weeks) (3). Although PE presents with diverse clinical features, severity, and outcomes, recent medical advances have made it possible to predict and prevent early-onset PE in many cases (4).

Traditionally, two models have been proposed for PE pathogenesis. The vascular model attributes PE to poor placental perfusion and oxidative stress, whereas the immunological model considers it a maladaptive maternal immune response to pregnancy. Both models emphasize a common feature: abnormal placentation, particularly inadequate spiral artery remodeling (5). However, these models often focus on events in the second trimester of pregnancy. Emerging evidence now points to even earlier origins, specifically, during implantation (6–10). Implantation is a highly regulated process that begins around days 6–10 post-fertilization and involves blastocyst apposition, adhesion, and invasion into the endometrial lining (7, 11). These early events establish the foundation for placental development, immune tolerance, and uteroplacental circulation (6, 12, 13).

Disruption of these processes, referred to as impaired implantation, can trigger a cascade of pathophysiological event (9, 10). These outcomes, impaired trophoblast invasion, inadequate spiral artery remodeling, and placental hypoxia, are key features of PE pathogenesis (8, 14–16). We propose that impaired implantation is not merely a consequence but may be an initiating event in PE development (8, 9, 17). Several recent high-quality reviews have examined placentation defects, decidualization resistance and immunology in PE (for example (18–21): While these works document evidence for an early maternal contribution, our review differs in three ways. First, we integrate the epidemiology of programmed frozen embryo transfer and corpus-luteum–mediated signaling with peri-conception exposure biology (including seminal-plasma interactions) to propose testable, mechanism-linked pathways. Second, we synthesize recent multi-omic and proteomic evidence for decidualization resistance and bring it into dialogue with immune receptor (e.g., KIR–HLA) and CD40–CD40L pathway data to suggest specific mechanistic experiments and translational biomarkers. Third, we translate these converging strands into a practical, prioritized research and prevention agenda (preconception cohorts, peri-implantation sampling, and candidate interventional strategies). In short, rather than reiterating that decidual dysfunction is important, this narrative explicitly maps distinct upstream implantation-era mechanisms to downstream clinical phenotypes and concrete study designs, offering a hypothesis-driven roadmap for validation.

## 2 Implantation

**Figure 1:**
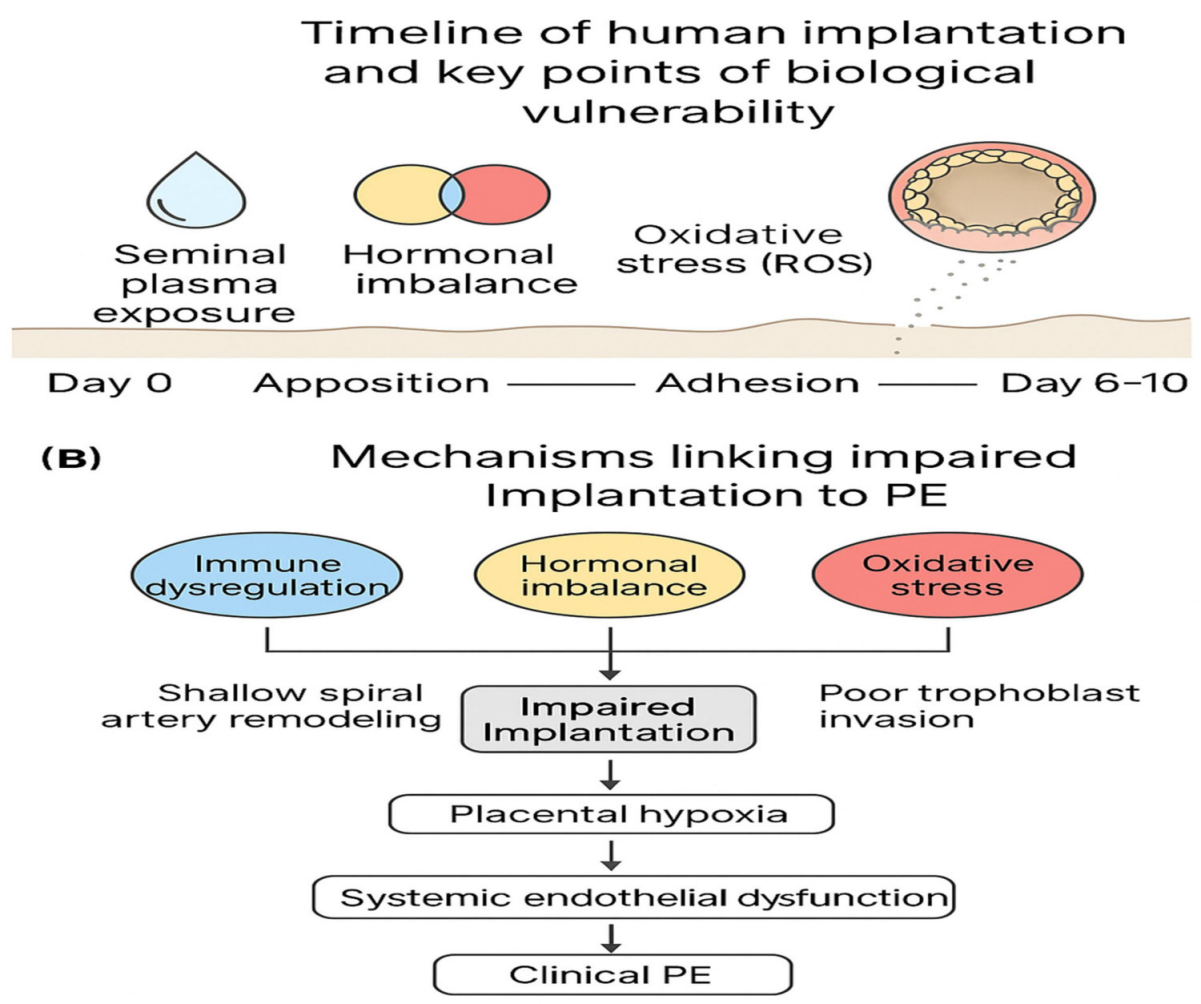
Timeline of implantation stages (apposition, adhesion, invasion) and major biological processes that may be vulnerable to disruption, including immune dysregulation, hormonal imbalance, and oxidative stress. These early disturbances can contribute to impaired implantation, leading to shallow spiral artery remodeling, placental hypoxia, and subsequent PE.

Implantation is a critical step in early pregnancy that determines the trajectory of embryonic development and placental formation (22). It begins approximately 6–10 days after fertilization and involves a sequence of well-orchestrated events, including apposition, adhesion, and invasion of the blastocyst into the endometrial lining (7, 12, 23). Figure 1 Timeline of implantation stages and key biological processes vulnerable to disruption. Early abnormalities, such as immune dysfunction, hormonal imbalance, and oxidative stress, may predispose to impaired implantation and future PE.

**Figure 2:**
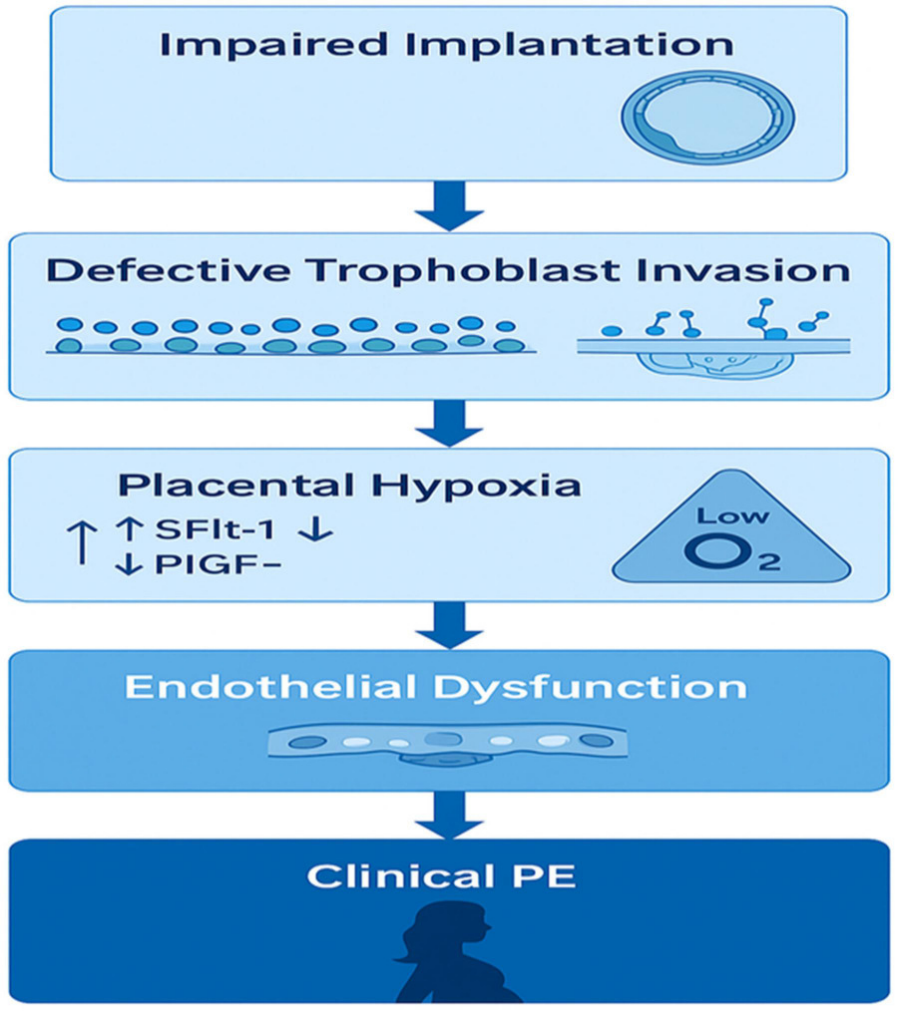
Simplified chronological pathway from impaired implantation to maternal syndrome A linear schematic showing the stepwise progression from impaired implantation → shallow trophoblast invasion → failed spiral artery remodeling → placental hypoxia → angiogenic imbalance → maternal endothelial dysfunction → clinical PE.

Successful implantation requires a receptive endometrium, functional trophoblasts, appropriate maternal immune modulation, and synchronized hormonal signaling, particularly estrogen and progesterone (11, 22, 24, 25). These processes ensure proper trophoblast differentiation and the establishment of a stable fetal-maternal interface (6, 13, 26). The window of implantation, a limited time frame during which the endometrium is optimally receptive, is regulated by a cascade of signaling pathways that include cytokines, growth factors, integrins, and steroid hormones (11, 27–29). Progesterone is central to decidualization, the transformation of stromal fibroblasts into decidual cells, which supports embryo acceptance and early immune tolerance (30, 31). Implantation is also heavily influenced by immunological factors. The maternal immune system undergoes a state of tolerance facilitated by local immune cell populations such as uterine natural killer (uNK) cells, macrophages, and regulatory T cells (32, 33). These cells modulate cytokine responses to protect the semi-allogeneic embryo from immune rejection while allowing for controlled inflammation necessary for implantation (25, 32–36). Disruption at any stage of this process, from hormonal dysregulation to immune maladaptation can lead to implantation failure, recurrent pregnancy loss, or defective placental development, all of which have been implicated in the pathogenesis of PE (8, 9, 37–44). To situate these biological events within the pathophysiological progression toward PE, we include a simplified chronological schematic (Figure 2) that illustrates the sequence from impaired implantation to shallow trophoblast invasion, angiogenic imbalance and ultimately the maternal syndrome.

## 3 Impaired implantation and its relevance to early pregnancy

**Figure 3:**
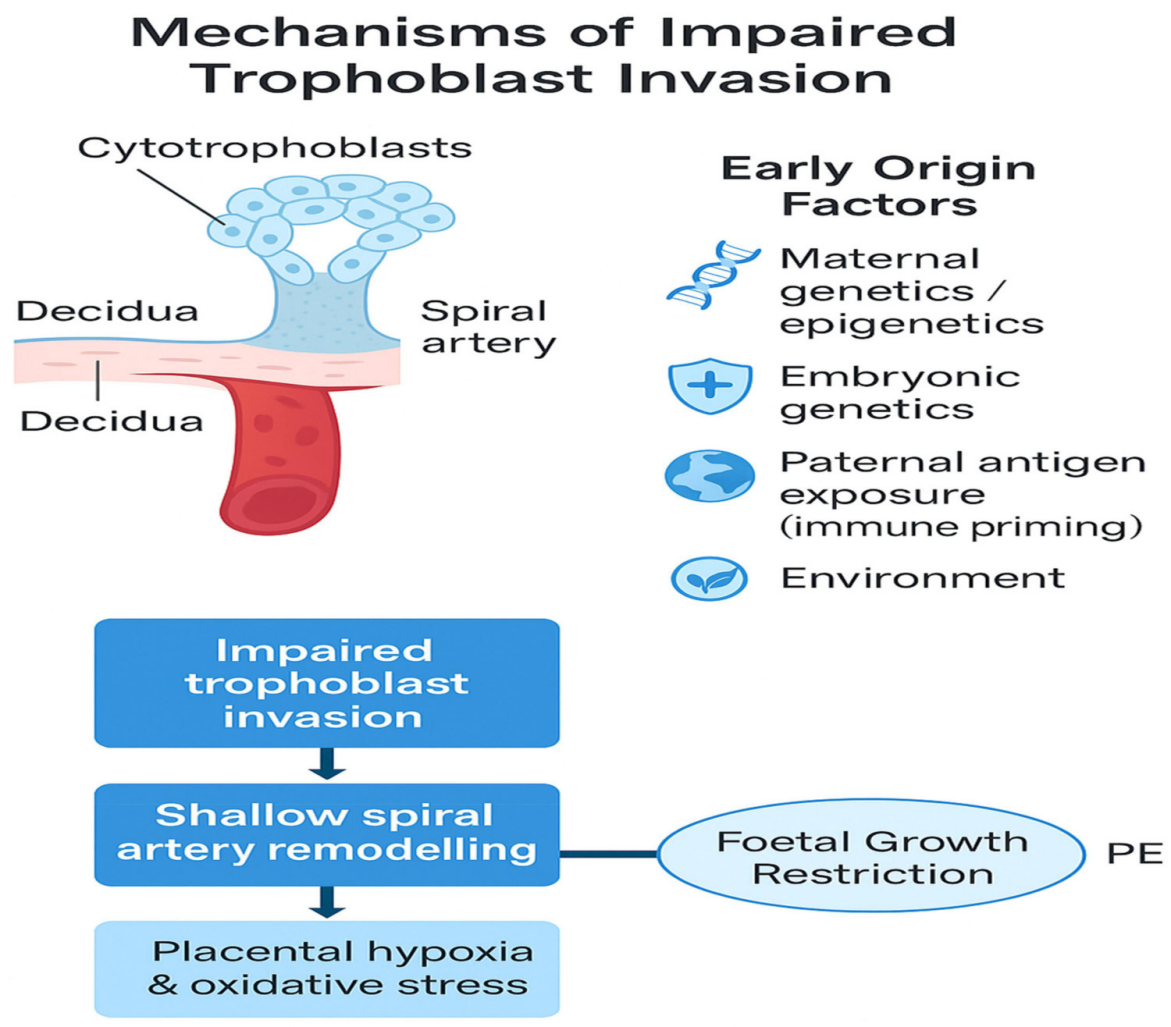
Central mechanisms linking impaired implantation to the development of PE. Impaired trophoblast invasion, immune imbalance, and defective spiral artery remodeling lead to placental hypoxia, systemic endothelial dysfunction, and the clinical syndrome of PE.

Impaired implantation refers to suboptimal blastocyst attachment, invasion, or integration into the endometrium (39, 40). Although traditionally discussed in the context of infertility and assisted reproduction, its relevance to spontaneous, pregnancies particularly in relation to complications such as PE, is increasingly recognized (8, 19). This is especially true regarding its link to obstetric complications such as PE (8, 11, 39, 44–46). Proper implantation ensures early immune tolerance, efficient nutrient exchange, and spiral artery remodeling, key elements for a healthy pregnancy (10, 12, 35). Disruption of any component in this process can lead to defective placentation, which is now considered a central mechanism underlying the development of PE (5, 8, 9). Recent studies suggest that implantation failure may precede and possibly cause impaired second-wave trophoblast invasion (8, 10), long thought to be the hallmark of PE. Rather than occurring mid-pregnancy, the pathogenic process may begin as early as the peri-implantation window, when immune signaling, hormonal priming, and cell adhesion dynamics are most active (10, 47, 48). For example, dysregulated decidualization due to impaired progesterone signaling or inflammatory insults leads to altered endometrial receptivity and abnormal embryo attachment (19, 30, 31, 49). Similarly, oxidative stress present during early implantation stages has been shown to reduce trophoblast viability and invasion capacity (50–52). Moreover, infections such as Chlamydia trachomatis (53) and viral agents like Zika virus (Tan et al., 2019) can disrupt epithelial integrity, immune balance, and implantation signaling pathways, providing further evidence of the sensitivity of early implantation to pathological insults (54–57). These disturbances can ultimately result in a deficient placental bed, an abnormal maternal-fetal interface, and systemic endothelial damage, the pathological triad of PE (9, 58, 59). These interconnected mechanisms are illustrated in Figure 3 Central mechanisms linking impaired implantation to the development of PE. Impaired trophoblast invasion, immune imbalance, and defective spiral artery remodeling lead to placental hypoxia, systemic endothelial dysfunction, and the clinical syndrome of PE.

As such, there is growing consensus that the implantation phase may represent the earliest point of therapeutic intervention to prevent adverse pregnancy outcomes (Moreno et al., 2023). Understanding its precise role in the initiation of PE could lead to novel diagnostics and interventions, targeting the uterine environment before clinical signs manifest (9, 10).

## 4 Immune dysregulation and the CD40–CD40L pathway in impaired implantation and potential trigger of PE

The maternal immune system must strike a delicate balance between tolerating the semi-allogeneic fetus and mounting appropriate defenses against pathogens (60, 61). During implantation, local immune modulation is essential to support trophoblast invasion and endometrial receptivity (25, 34, 35). Failure to establish this immunological equilibrium can disrupt implantation, setting the stage for placental insufficiency and PE (49, 59, 62).

One of the pivotal players in immune-mediated implantation failure is the CD40–CD40L (CD154) pathway (17). The interaction between CD40 on stromal cells and CD40L, mainly expressed on activated T cells and platelets, promotes the release of proinflammatory cytokines such as IL-6, TNF-α, and IFN-γ (63–65). These cytokines impair decidualization and disturb the immune-tolerant uterine microenvironment critical for early embryo development (17, 63). Animal studies have confirmed the role of CD40L-induced inflammation in triggering PE-like symptoms. In murine models, overactivation of this pathway during the implantation window results in defective spiral artery remodeling, placental hypoxia, and elevated maternal blood pressure, hallmarks of PE (17, 66). Emerging evidence shows link between PE and CD40L in humans (65, 67, 68) Moreover, excessive stimulation of this pathway may lead to the recruitment of cytotoxic NK cells and effector T cells, disturbing the local Treg–Th1/Th17 balance required for fetal tolerance (62, 69). Comprehensive reviews have argued that failure to induce adequate maternal immune tolerance, particularly impaired expansion of regulatory T cells and dysregulated antigen presentation, predisposes to shallow trophoblast invasion and the later maternal syndrome of PE (70). This inflammatory imbalance, when sustained, leads to trophoblast apoptosis, shallow invasion, and a dysfunctional maternal-fetal interface, making the uterine environment increasingly hostile (71, 72).

Thus, immune dysregulation, especially involving the CD40–CD40L axis, represents a crucial mechanistic link between implantation failure and the pathogenesis of PE (17). Targeting this pathway may offer novel opportunities for preventive immunomodulatory therapies in early pregnancy.

## 5 Oxidative stress at the implantation–placentation interface

Oxidative stress (OS) has emerged as a major contributor to pregnancy complications (73), particularly PE (50–52, 74). It refers to an imbalance between the generation of reactive oxygen species (ROS) and the body's ability to counteract their harmful effects with antioxidants (73). While OS has been extensively studied in the context of placental dysfunction later in pregnancy, mounting evidence suggests that oxidative damage begins much earlier, during implantation and early placentation (51, 74–78). During the implantation window, physiological oxidative stress is required for trophoblast differentiation, angiogenesis, and decidual remodeling (14, 79, 80). However, when ROS levels exceed physiological limits, due to maternal conditions like obesity, infections, or environmental exposures, these same processes are disrupted. Excessive ROS during early pregnancy impairs blastocyst-endometrium cross-talk, inhibits trophoblast proliferation, and reduces vascular endothelial growth factor (VEGF) expression, resulting in poor vascularization and defective spiral artery remodeling (52, 81, 82).

Oxidative stress also triggers apoptosis of cytotrophoblasts and syncytiotrophoblasts, further weakening placental development and nutrient exchange (51, 83, 84). As a result, the placenta becomes ischemic, setting off a feedback loop of inflammation, hypoxia, and systemic endothelial dysfunction, key mechanisms in PE pathogenesis (80, 81, 85, 86). Furthermore, ROS can modify gene expression in endometrial cells, alter immune tolerance, and interfere with decidualization, creating a hostile uterine environment even before clinical signs of PE emerge (51, 87–89). Elevated OS biomarkers during the first trimester are increasingly being explored as predictive tools for PE, offering a promising avenue for early risk stratification (73, 75, 90, 91). Thus, oxidative stress at the implantation–placentation interface serves not merely as a downstream effect of placental dysfunction, but as a primary initiator of the pathological cascade leading to PE. Addressing oxidative imbalance in early pregnancy could be key to preventing the onset of PE.

## 6 Seminal plasma and paternal antigen tolerance in implantation success and PE risk

Seminal plasma (SP) is increasingly recognized as a critical biological fluid in reproduction, extending beyond its classical role in sperm transport to actively modulate the maternal immune system (92, 93). Rich in cytokines, prostaglandins, extracellular vesicles, and immunomodulatory proteins, SP initiates complex signaling cascades in the female reproductive tract that are crucial for successful implantation and maternal immune tolerance (93–95). Following insemination, SP triggers a controlled inflammatory response characterized by leukocyte infiltration (notably neutrophils and macrophages), upregulation of cytokines such as IL-6, IL-8, GM-CSF, and the recruitment and activation of antigen-presenting cells (96–99). These processes promote the expansion of regulatory T cells (Tregs) and the establishment of maternal tolerance toward paternal alloantigens expressed by the embryo (100, 101). Exposure to SP-derived transforming growth factor-β (TGF-β) and soluble HLA-G molecules is particularly pivotal in skewing the uterine immune environment toward tolerance, facilitating endometrial remodeling, decidualization, and vascular adaptation necessary for blastocyst implantation (102, 103). However, the timing and composition of SP exposure are critical determinants of its effects. Mistimed SP exposure, particularly during the peri-implantation window may overstimulate uterine inflammation, leading to impaired trophoblast invasion, shallow implantation, and an elevated risk of pregnancy complications, including PE (104–106). Prostaglandins present in SP, especially PGE₂, play dual roles: promoting angiogenesis and immune tolerance at physiological levels, but exacerbating neutrophil chemotaxis and local cytokine surges when dysregulated (107–109). Excessive pro-inflammatory signaling during the window of implantation can disrupt the delicate balance needed for embryo acceptance, compromising placentation and triggering a cascade leading to endothelial dysfunction characteristic of PE (10, 49, 80, 104, 110).

**Figure 4:**
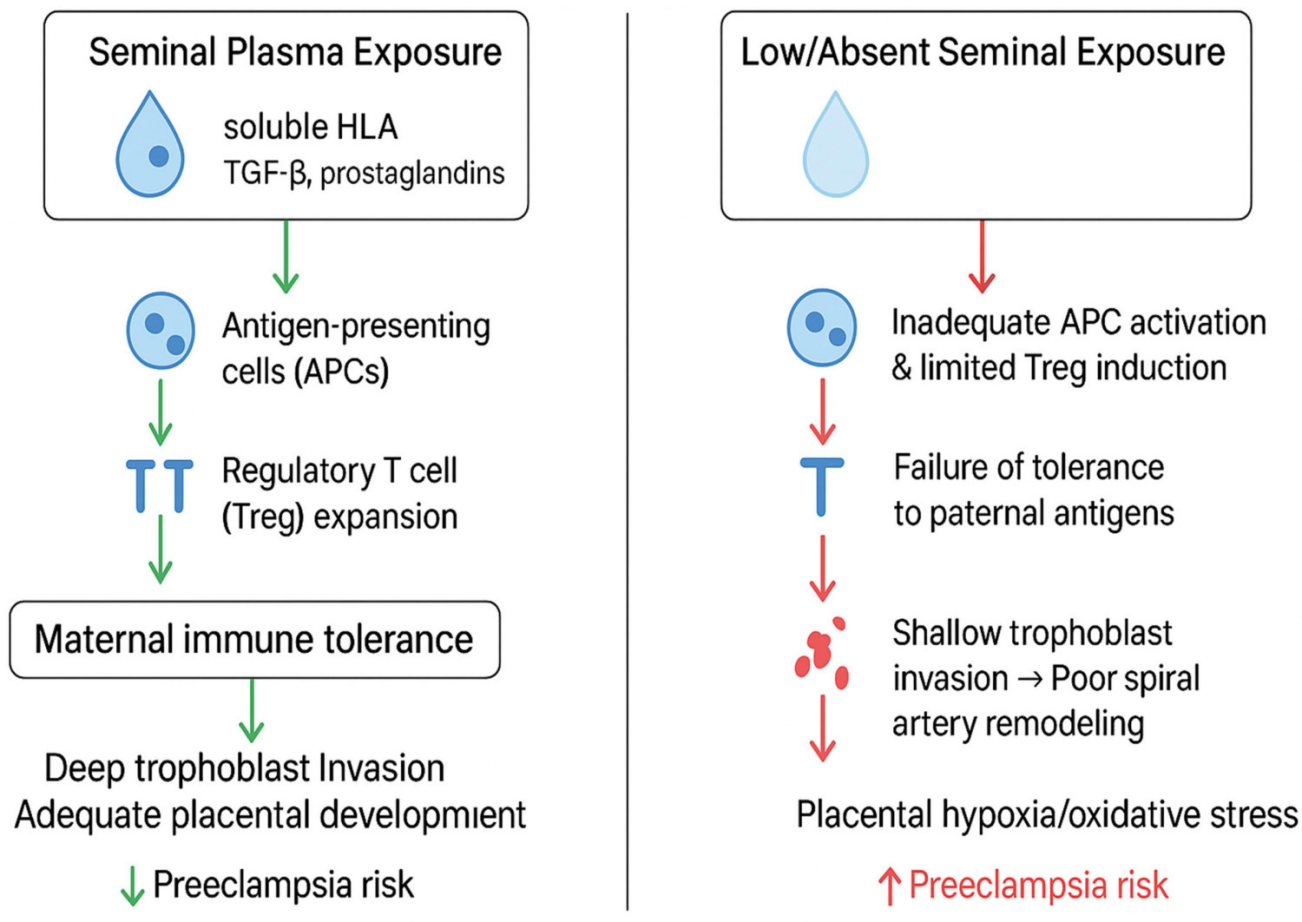
Role of seminal plasma exposure in shaping maternal immune tolerance and PE risk. (Left) Adequate seminal plasma exposure delivers soluble HLA, TGF-β, and prostaglandins to the maternal reproductive tract, stimulating antigen-presenting cells and expanding regulatory T cells, leading to immune tolerance, deep trophoblast invasion, and reduced risk of PE. (Right) Low or absent exposure results in inadequate tolerance, shallow trophoblast invasion, poor spiral artery remodeling, placental hypoxia, and increased PE risk. Based on Koelman et al. (113), Triche et al. (154), Saito et al. (70), Emmery et al. (146), and Almasi-Hashiani et al. (181).

Interestingly, epidemiological data show that limited exposure to a partner's SP, such as through barrier contraception or short sexual relationships before conception, correlates with an increased risk of PE (105, 111, 112). Early epidemiological studies found that greater exposure to a partner's seminal fluids (including oral exposure) was associated with a reduced risk of PE; Koelman et al. proposed that soluble HLA molecules in seminal plasma may mediate maternal immune conditioning prior to implantation (113). Analyses stratified by duration of sexual cohabitation show that longer preconception exposure to paternal sperm is associated with decreased PE risk, consistent with antigenic tolerance hypotheses (114). This suggests that SP-mediated maternal immune conditioning prior to conception is protective, while inappropriate immune activation during implantation is pathological. As illustrated in Figure 4 seminal plasma exposure represents a double-edged sword in shaping maternal immune responses and implantation outcomes. On the protective side (left panel), adequate exposure delivers soluble HLA molecules, TGF-β, and prostaglandins to the maternal reproductive tract. These signals activate antigen-presenting cells, expand regulatory T cells, and promote maternal immune tolerance, leading to deep trophoblast invasion, effective spiral artery remodeling, and reduced risk of PE. Conversely (right panel), limited or absent exposure results in poor immune priming, inadequate tolerance to paternal antigens, and shallow trophoblast invasion. This cascade contributes to placental hypoxia, oxidative stress, and ultimately increases the risk of PE. Together, these pathways highlight how both immunogenetic compatibility and behavioral factors such as semen exposure can critically determine implantation success and disease risk.

Thus, seminal plasma represents a double-edged sword in reproductive immunology. Adequate preconception exposure fosters maternal immune adaptation and reduces PE risk (112, 115), while excessive or poorly timed exposure during implantation may heighten inflammatory responses, impair implantation, and precipitate PE (105). Understanding these mechanisms offers novel insights into the early events that predispose to PE and opens avenues for preventive interventions in high-risk populations.

## 7 Hormonal and molecular disruptions during implantation

Hormonal signaling between the embryo and the endometrium is pivotal for successful implantation (46, 116). Progesterone and estrogen orchestrate endometrial receptivity, regulate immune tolerance, and modulate trophoblast invasion (7, 31, 117, 118). However, hormonal resistance or dysregulation can disturb these finely tuned interactions, contributing to implantation failure and, subsequently, PE (119, 120). Progesterone resistance, characterized by an impaired response of endometrial cells to circulating progesterone, has been increasingly implicated in impaired decidualization and suboptimal implantation (19, 31). Progesterone receptor-A (PR-A) expression in endometrial epithelial cells declines during the “window of receptivity,” facilitating the transition to a receptive state (31, 121, 122)). Disruption of this downregulation, either through intrinsic defects or inflammatory insults, can impair epithelial receptivity and hinder blastocyst attachment.

In parallel, defects in key molecular pathways involved in implantation have been described. Aberrant expression of adhesion molecules (e.g., integrins, selectins) and extracellular matrix components impairs embryo-endometrial interactions (22, 123–125). Reduced expression of cytokines such as leukemia inhibitory factor (LIF) and HOXA10 also correlates with implantation failure and adverse pregnancy outcomes (126–128). Furthermore, hormonal disturbances exacerbate oxidative stress at the implantation site, as progesterone deficiency increases ROS accumulation, further compromising trophoblast function and endometrial receptivity (87, 129, 130). Thus, hormonal and molecular disruptions during the critical window of implantation undermine immune adaptation, vascular remodeling, and trophoblast differentiation, establishing conditions that predispose to placental insufficiency and the later development of PE.

## 8 Infections and environmental toxins affecting implantation

Infections and environmental exposures during early pregnancy can profoundly disrupt implantation by triggering inflammatory responses, altering endometrial receptivity, and damaging trophoblast function (56, 77, 131). Several pathogens have been implicated in impaired implantation. For instance, Chlamydia trachomatis infection of endometrial stromal cells induces defective decidualization and aberrant chemokine release, leading to an inhospitable uterine environment (53). Viral infections, notably Zika virus, have been shown to target trophectoderm cells during the peri-implantation window, impairing implantation and subsequent placental development (54). Environmental toxins also pose significant threats. Cigarette smoking and exposure to tobacco-related toxins increase oxidative stress, impair angiogenesis, and inhibit decidual transformation (132). Alcohol consumption during the periconceptional period disrupts vascular endothelial growth factor (VEGF) signaling at the maternal-fetal interface, contributing to early placental dysfunction (6, 133). Endocrine-disrupting chemicals, such as phthalates and bisphenol A, disturb steroid hormone signaling and may promote inflammatory activation within the endometrium (134). Moreover, psychological stress, another environmental factor, can elevate systemic cortisol levels, suppress progesterone-mediated endometrial receptivity, and provoke immune dysregulation, further compromising implantation success (78, 135, 136).

Collectively, infections and environmental insults impair implantation by promoting local inflammation, increasing oxidative stress, and disrupting molecular signaling critical for trophoblast invasion and decidualization. These early disturbances set the foundation for defective placentation and increase the risk of PE and other adverse pregnancy outcomes.

## 9 Genetic and immunological risk factors for implantation failure

Genetic and immunological factors play critical roles in regulating implantation success and, consequently, influence the risk of developing PE. Disturbances in immune recognition and genetic compatibility between the mother and fetus can impair maternal-fetal tolerance, leading to implantation failure and defective placentation (34, 60, 137–140). One significant mechanism involves human leukocyte antigen (HLA) incompatibility. The trophoblast expresses non-classical HLA molecules, such as HLA-G, that modulate maternal immune responses and promote tolerance (103, 140–142). Couples with certain HLA polymorphisms have a higher risk of recurrent pregnancy loss and impaired implantation, suggesting that genetic mismatches between mother and father can contribute to early pregnancy failure and PE development (137, 143–145). Genetic studies have identified associations between particular maternal and fetal HLA class I/II alleles and severe PE, further implicating maternal–fetal immunogenetic interactions in disease risk (146). Uterine natural killer (uNK) cells, abundant in the decidua during early pregnancy, recognize trophoblast HLA molecules. Dysregulation of uNK cell function, through excessive activation or defective inhibition, has been associated with poor spiral artery remodeling and increased risk of PE (33, 34, 147–149). Another important immune mechanism is the expansion of regulatory T cells (Tregs) (150). Seminal plasma exposure before conception induces Treg differentiation, promoting tolerance toward paternal antigens (151–153). Insufficient Treg expansion has been linked to implantation failure, miscarriage, and PE (Robertson et al., 1997; Schjenken et al., 2021). A case–control study of nulliparous women found that maternal–fetal HLA sharing was associated with increased odds of PE, and that this association was stronger among women with limited prior vaginal exposure to the father's seminal fluid, supporting an interaction between immunogenetic compatibility and seminal-fluid-mediated immune priming (154).

Additionally, maternal thrombophilias, such as Factor V Leiden mutation and antiphospholipid syndrome, predispose women to defective implantation by promoting microthrombi formation within the developing placenta (155). These genetic conditions disrupt early placental perfusion, contributing to oxidative stress, trophoblast dysfunction, and the later emergence of PE (156–161).

## 10 Antiphospholipid syndrome (APS) and immune-mediated endothelial/trophoblast injury

Antiphospholipid syndrome (APS) is clinically associated with a substantially increased risk of early-onset PE and other placental-mediated complications (162). Anti-β2-glycoprotein I (anti-β2GPI) antibodies bind β2-GPI on endothelial and trophoblast surfaces, promote β2-GPI dimerisation and enable functional engagement of the ApoER2/LRP8 receptor (163). ApoER2 activation triggers a Dab2/PP2A-dependent signalling cascade that reduces endothelial nitric oxide synthase (eNOS) phosphorylation and nitric-oxide availability, producing a pro-adhesive, pro-inflammatory endothelial phenotype (164). Recent endothelial studies further demonstrate that this anti-β2GPI–LRP8 signalling is lipid-raft–dependent and suppresses eNOS phosphorylation through LRP8 activation in raft domains (165). *In vivo* and *in vitro* trophoblast experiments show that ApoER2 also mediates aPL-induced trophoblast dysfunction and pregnancy complications, directly linking this pathway to impaired trophoblast invasion and differentiation (166). Additional trophoblast studies demonstrate that aPL reduce trophoblast migration and invasion and amplify complement-mediated decidual inflammation (167). Intraplacental microthrombosis observed in APS adds another upstream mechanism through which spiral-artery remodelling may be disrupted (167). Collectively, these endothelial and trophoblast effects position APS as a paradigmatic immune-mediated upstream cause of implantation-era defects that predispose to placental hypoxia, angiogenic imbalance and the maternal endothelial syndrome of PE.

## 11 Cross-species and evolutionary evidence of PE-like disorders

Hemochorial placentation, characterized by deep trophoblast invasion into the maternal decidua, is unique to humans and a few primate species (16, 168, 169). While this adaptation enhances nutrient and gas exchange to support larger-brained offspring, it also introduces significant immunological and vascular challenges that increase the risk for pregnancy disorders such as PE (13, 169, 170). Observations in non-human primates further support the role of implantation defects in the pathogenesis of PE. Cases of PE-like syndromes have been reported in patas monkes and chimpanzees (171–173). These spontaneous occurrences mirror human PE symptoms, including hypertension, proteinuria, and fetal distress, suggesting a common evolutionary vulnerability tied to the demands of invasive placentation. Evolutionarily, the maternal immune system has adapted to tolerate the semi-allogeneic fetus through mechanisms such as expansion of uterine natural killer (uNK) cells and regulatory T cells (Tregs) (34). However, when these adaptations are insufficient or disrupted—whether by genetic incompatibility, environmental exposures, or infections, the risk of impaired implantation and subsequent placental dysfunction increases.

**Figure 5:**
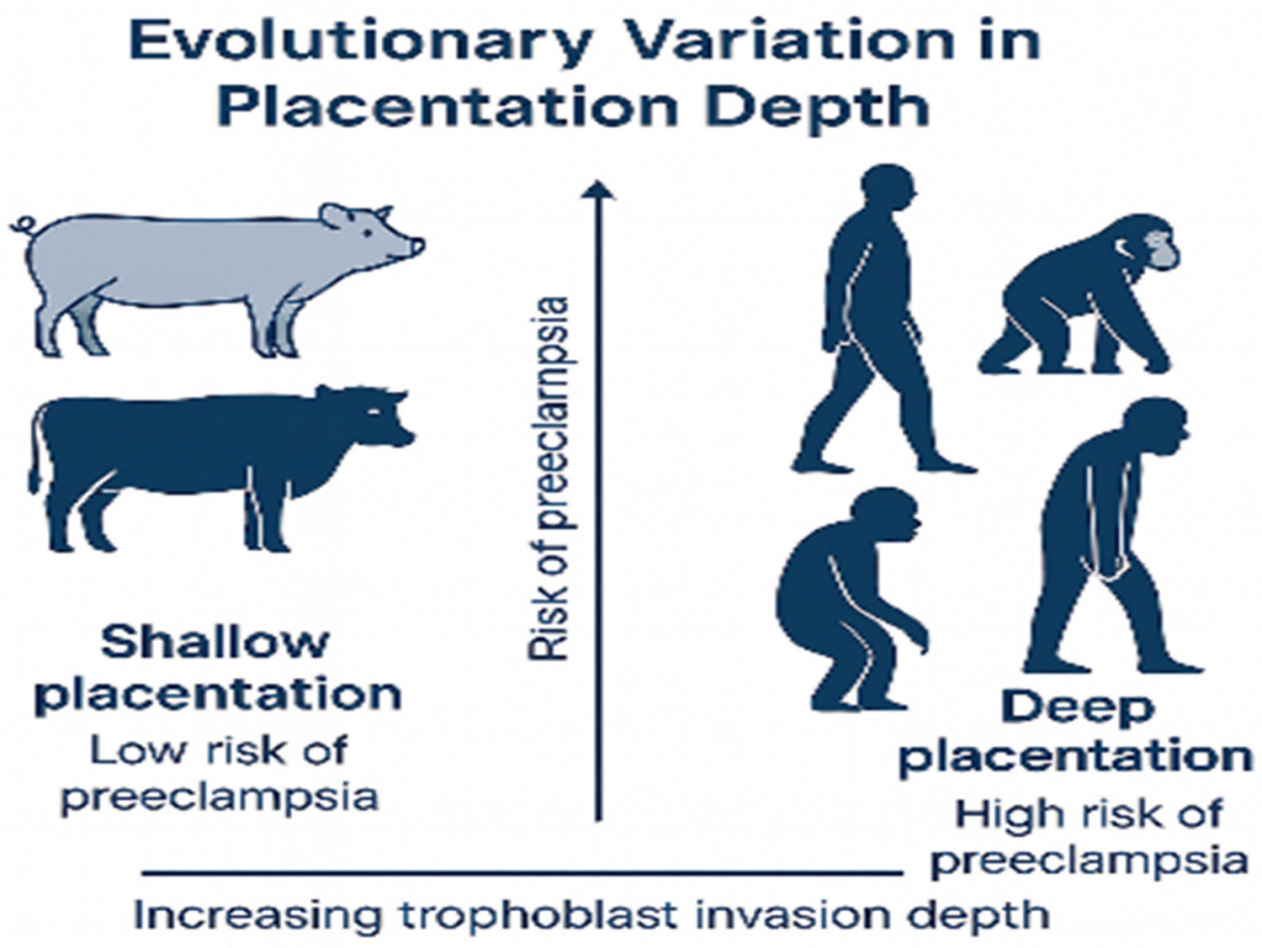
Evolutionary differences in placentation depth among mammals. Species with deep trophoblast invasion (e.g., humans, gorillas) are at higher risk for PE, while those with shallow placentation (e.g., pigs, cows) exhibit minimal risk.

Comparative studies reveal that the depth of trophoblast invasion correlates with pregnancy risk: species with shallow implantation (e.g., pigs, cows) rarely experience PE-like complications, whereas those with deep invasion (e.g., humans, gorillas) are more vulnerable Figure 5 (168, 174–176).

This biological pattern reinforces the hypothesis that the success or failure of early implantation events critically determines placental health and pregnancy outcome. Thus, evolutionary and cross-species evidence underscores that early implantation failure, rather than second-trimester vascular dysfunction alone, constitutes the foundational defect in PE. PE may not solely stem from deficiencies in the second wave of trophoblast invasion. Instead, it posits that the initial impaired implantation of the blastocyst acts as the primary trigger, initiating a cascade of events that ultimately influence the depth of trophoblast invasion (170, 177, 178). This early implantation defect may result in superficial trophoblast invasion, a hallmark of PE pathogenesis.

## 12 Clinical and epidemiological evidence linking implantation failure to PE

Clinical and epidemiological findings suggest that early implantation abnormalities may underlie a subset of PE cases (8, 10, 169). Early disturbances in uterine vascularization, immune tolerance, and trophoblast invasion are increasingly recognized as predictors of later pregnancy complications (6, 179, 180).

A large meta-analysis found that pregnancies conceived via ART have approximately a 1.7-fold higher risk of PE than spontaneous conceptions, with particularly high risks observed in programmed frozen embryo transfer cycles, supporting the role of periconceptional hormonal/endometrial environment in later placental dysfunction (181). Large cohort analyses report substantially higher adjusted odds of hypertensive disorders, including PE, after programmed (artificial cycle) frozen embryo transfer compared with fresh transfer or natural-cycle FET, e.g., an adjusted OR ≈ 2.4 for hypertensive disorders after artificial-cycle FET in a large multicenter analysis, supporting a role for corpus luteum absence or altered peri-conceptional endocrine/immune milieu in the pathogenesis (182).

Assisted reproductive technologies (ART) offer a unique model to study implantation dynamics (183). Women who conceive via *in vitro* fertilization (IVF) exhibit a higher incidence of PE compared to those with spontaneous conceptions (184, 185). Particularly, embryos transferred in non-physiological hormonal environments show increased risks, suggesting that suboptimal implantation conditions, independent of genetic factors, can predispose to placental dysfunction (18, 183, 184, 186, 187). Early uterine artery Doppler studies further support a link between implantation failure and PE. Abnormal uterine artery pulsatility indices observed in the first trimester correlate with impaired trophoblast-mediated spiral artery remodeling and are predictive of later PE development (188, 189). These vascular anomalies appear well before clinical manifestations, highlighting the importance of early implantation events in disease initiation.

**Figure 6:**
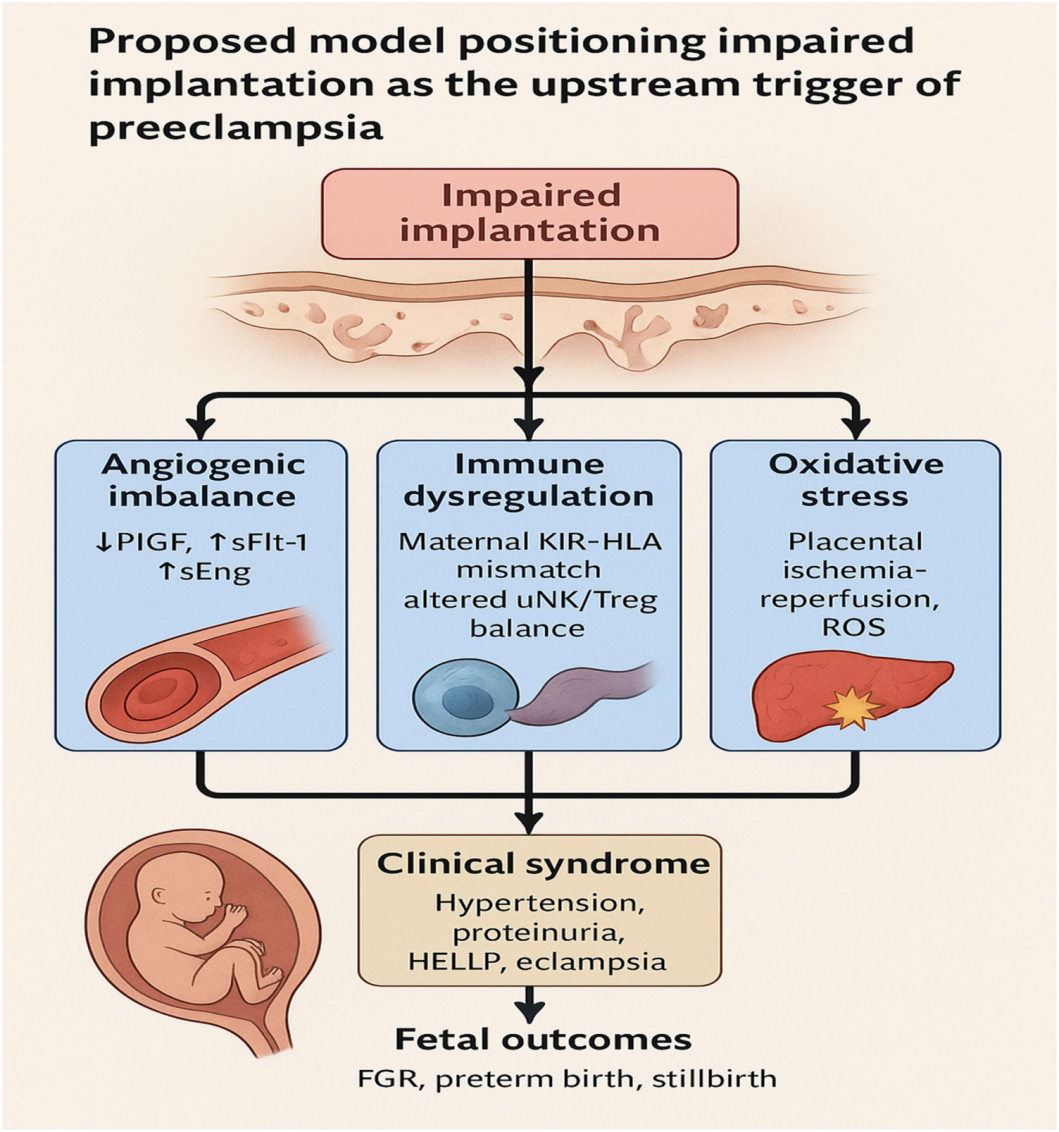
Proposed pathophysiological model of PE. Impaired implantation, characterized by defective decidualization and shallow trophoblast invasion, is positioned as the upstream initiating event. This defect triggers downstream amplifiers including angiogenic imbalance (↓PlGF, ↑sFlt-1, ↑sEng), immune dysregulation (maternal KIR–HLA mismatch, altered uNK/Treg balance), oxidative stress (placental ischemia–reperfusion, ROS generation), and maternal constitutional risk factors (chronic hypertension, diabetes, obesity, advanced age). These pathways converge on systemic endothelial dysfunction, leading to maternal complications (hypertension, proteinuria, HELLP syndrome, eclampsia) and adverse fetal outcomes (fetal growth restriction, preterm birth, stillbirth). The model reframes PE as a disorder rooted in early implantation failure, with secondary modifiers shaping disease severity and onset.

Epidemiological studies suggest an association between limited paternal antigen exposure and increased PE risk, although causal inference remains uncertain. Women using barrier contraception or with short sexual relationships before conception show higher rates of PE, implying that insufficient maternal immune priming against paternal antigens may compromise implantation and vascular adaptation (105, 112, 115, 190, 191). Additionally, recurrent implantation failure (RIF) in the context of infertility is associated with an increased risk of adverse obstetric outcomes, including PE and fetal growth restriction (44, 192, 193). These observations suggest that failure of embryo attachment and immune adaptation can have lasting consequences on placental function and maternal health. The diverse biological pathways described above can be integrated into a unifying framework, with impaired implantation as the initiating trigger as illustrated in Figure 6.

Taken together, these mechanistic insights emphasize that PE is multifactorial, involving abnormalities in implantation, angiogenic signaling, immune regulation, oxidative stress, and maternal constitutional predisposition. While these pathways converge on impaired placentation as a central outcome, they differ in their upstream drivers and downstream clinical manifestations. To provide a consolidated overview, Table 1 Mechanistic pathways implicated in PE and candidate first-trimester biomarkers summarizes the major mechanistic pathways, their supporting evidence base, and potential first-trimester biomarkers, offering a framework that bridges mechanistic understanding with early diagnostic strategies.

**Table 1:** Mechanistic pathways implicated in PE and candidate first-trimester biomarkers.

Mechanism	Evidence type	Key refs	Evidence strength	Notes	Candidate first-trimester biomarkers
Impaired implantation/decidualization failure	Human observational studies, animal models, bioinformatics	(194)	Moderate	Inadequate decidualization and shallow trophoblast invasion precede clinical disease; prospective human validation limited	Uterine artery Doppler PI, decidual markers (IGFBP-1, prolactin), PAPP-A
Angiogenic imbalance (↑sFlt-1, ↓PlGF, ↑sEng)	Large cohorts, biomarker trials	(195–197)	Strong	Validated biomarkers precede onset; sFlt-1/PlGF ratio clinically adopted for short-term PE prediction	PlGF (↓), sFlt-1 (↑), sFlt-1/PlGF ratio
Immune dysregulation (KIR–HLA, uNK, Tregs)	Genetic association studies, functional immunology, animal models	(198, 199)	Moderate	KIR–HLA interactions reproducibly influence risk; larger, multiethnic cohorts needed	Maternal Treg frequency (FOXP3+), decidual uNK phenotype, cytokines (IL-10, TGF-β)
Oxidative stress (ROS, ischemia–reperfusion)	Placental analyses, mechanistic models, reviews	(50)	Strong	Widely accepted driver of placental dysfunction; clinical predictive thresholds unsettled	8-iso-PGF2α, MDA, antioxidant enzymes (SOD, GSH), cf-mtDNA
Seminal plasma/paternal exposure (immune priming)	Case–control and cohort studies	(105)	Weak–Moderate	Cumulative exposure protective; confounding factors remain; mechanistic human data limited	Cervicovaginal cytokines (TGF-β, IL-10), Treg induction, anti-paternal HLA antibodies
Assisted reproduction/FET cycles	Meta-analyses, multicenter cohorts	(200)	Strong	Programmed FET (no corpus luteum) consistently increases PE risk; mechanistic link to missing luteal factors	Serum progesterone, relaxin, PlGF, uterine artery PI
Maternal infection (UTI, periodontal, viral)	Observational studies, meta-analysis	(201)	Moderate	Modest risk increase with UTI and periodontal disease; heterogeneity across pathogens	Pathogen PCR, CRP, placental inflammatory markers
Maternal constitutional risk (HTN, diabetes, obesity, age)	Large epidemiological cohorts, guidelines	(202)	Strong	Established non-placental risk factors; consistently elevate baseline risk independently of implantation	Risk scores, BP, HbA1c, lipid profile, PlGF

## 13 Counterarguments and alternative models

Some argue that defective spiral artery remodeling, not implantation, remains the true initiating event in PE. This classical model has been influential for decades; however, it cannot fully account for epidemiological associations with assisted reproduction technologies, paternal seminal exposure, or the predictive performance of first-trimester biomarkers. These gaps suggest that implantation defects may represent an upstream cause, with abnormal spiral artery remodeling emerging as a downstream manifestation rather than the primary trigger.

Any complete model of PE must confront and integrate established alternative explanations rather than ignore them. The most widely accepted framework remains the two-stage (placental) model: deficient placentation (stage 1) produces a stressed placenta that releases factors into the maternal circulation and precipitates the maternal syndrome of hypertension, proteinuria and end-organ dysfunction (stage 2). This model explains a large body of histopathological and clinical data and remains the foundation for much current research into PE (203). A closely related and clinically useful account is the angiogenic-imbalance model. Excess circulating soluble fms-like tyrosine kinase-1 (sFlt-1) with reduced placental growth factor (PlGF) is strongly associated with the later maternal syndrome, and the sFlt-1/PlGF ratio now has validated short-term predictive and diagnostic utility in symptomatic women (204). These angiogenic biomarkers are already used in practice to help risk-stratify and manage suspected PE (205).

Third, maternal constitutional factors, chronic hypertension, obesity, diabetes, and preexisting cardiovascular or renal disease, clearly raise baseline PE risk and can precipitate the syndrome with less placental injury than otherwise required (18). In other words, maternal susceptibility can act independently of, or interact with, placental origins to determine whether and when clinical PE appears.

Genetic and immunogenetic findings add nuance rather than simple contradiction. Maternal KIR and fetal HLA-C combinations modulate uterine NK cell behavior and have been reproducibly associated with altered risk of PE and other adverse outcomes (198, 199); this work underlines that immunogenetic compatibility at the maternal–fetal interface is an important determinant of placentation quality.

**Figure 7:**
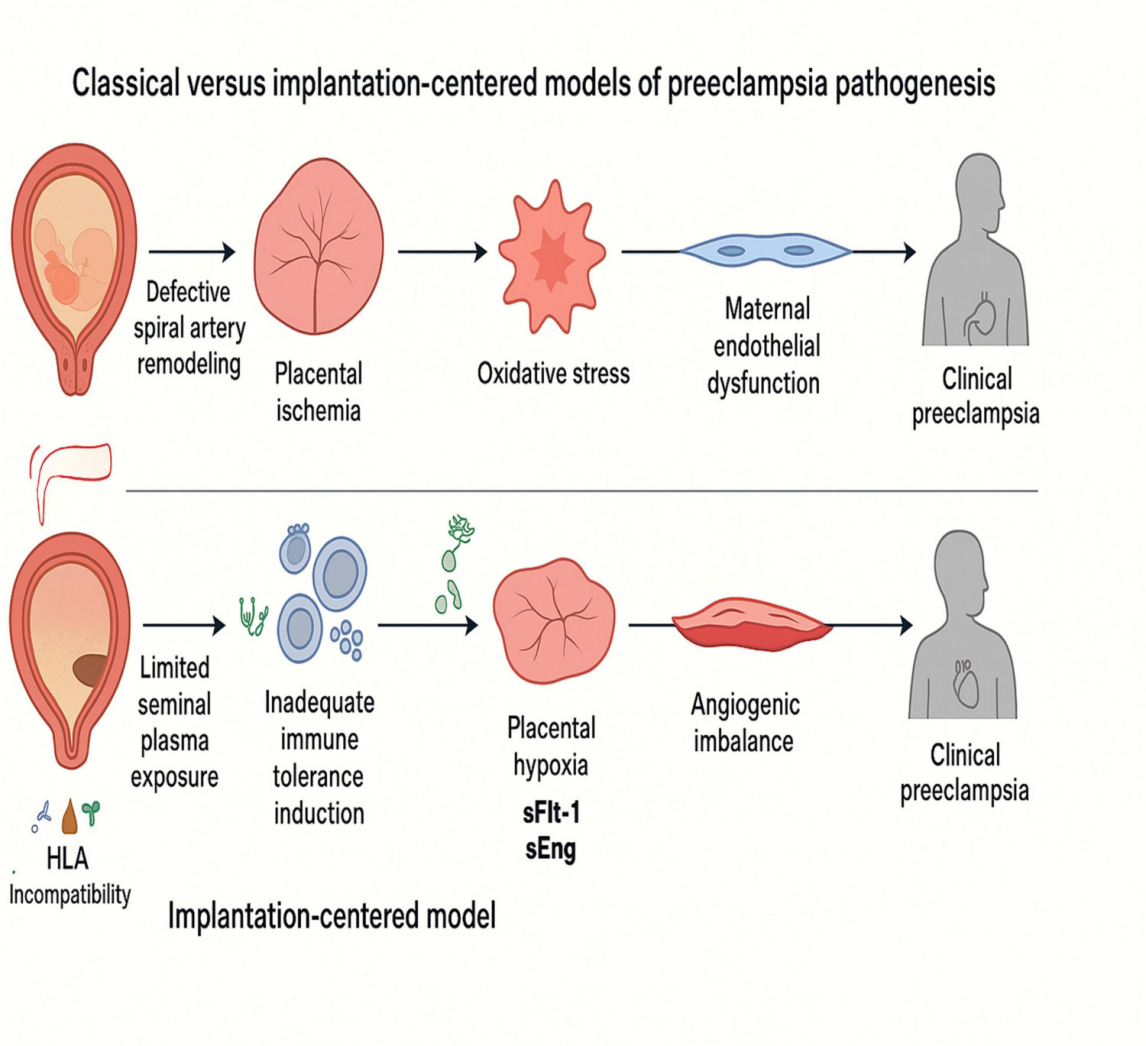
This illustration contrasts the classical two-stage model, which attributes PE to defective spiral artery remodeling and subsequent placental ischemia, with the implantation-centered model, which proposes that disease originates at the periconceptional stage. In the latter, limited seminal plasma exposure, maternal–fetal HLA incompatibility, and disrupted endocrine environments impair immune tolerance and decidualization, leading to shallow trophoblast invasion, placental hypoxia, angiogenic imbalance, and the maternal clinical syndrome.

Epidemiological observations that sometimes motivate non-implantation explanations should also be acknowledged. For example, assisted reproductive technologies, particularly certain frozen embryo transfer protocols and cycles lacking a corpus luteum, are associated with higher PE risk (206, 207), pointing to procedural or hormonal factors that may act independently of embryo quality. Likewise, observational studies linking limited seminal-plasma/sperm exposure or short cohabitation with higher PE risk support the immune-priming hypothesis (105, 114), but these associations are subject to confounding by sexual behavior, infection history, parity, and ART use and therefore cannot prove causality on their own. The implantation-centered framework challenges the prevailing two-stage model. Figure 7 contrasts the temporal sequence of events proposed in both paradigms, highlighting how implantation defects precede the angiogenic and immune abnormalities classically attributed to second-trimester placentation failure.

How these models fit with the implantation hypothesis. Rather than treating the implantation hypothesis as a rival that must displace present models, the parsimonious and testable position is that impaired implantation often acts upstream of the molecular and maternal pathways emphasized by the two-stage and angiogenic models. In this integrative view, implantation defects (immune, hormonal, or structural) can lead to shallow trophoblast invasion and suboptimal spiral-artery remodeling, which then provokes placental stress, dysregulated angiogenic signaling (sFlt-1/PlGF), and finally the maternal syndrome, with maternal constitutional factors modulating sensitivity at every step. In other cases, maternal disease or ART practice could dominate the causal chain and produce PE even when implantation itself is not the primary insult. The mechanisms described above illustrate the multifactorial origins of PE, ranging from impaired implantation and immune maladaptation to angiogenic imbalance, oxidative stress, assisted reproduction protocols, infections, and maternal constitutional risk factors. Each pathway contributes differentially to disease onset and progression, and their relative importance likely varies across patient subgroups.

## 14 Early biochemical and biophysical markers

Early biochemical and biophysical abnormalities are detectable in the peri-implantation and first-trimester window and precede clinical PE (208). A 2025 meta-analysis including >33,000 pregnancies found that first-trimester PAPP-A levels were significantly lower in pregnancies that later developed PE (MD ≈ −0.17 normalised units; early-onset PE MD ≈ −0.24), supporting the presence of measurable biochemical differences in the peri-implantation/early first-trimester window (209). PAPP-A alone has modest predictive performance but contributes meaningfully when combined with other biomarkers and maternal clinical factors.

Placental growth factor (PlGF) is reduced in early pregnancy in pregnancies destined for PE and adds complementary predictive information to PAPP-A (210).

Although sFlt-1/PlGF dynamics are most informative later in the second and third trimesters, their robust association with clinical PE provides a mechanistic bridge between early placental dysfunction and overt disease; dynamic prediction models and clinical reviews confirm the ratio's prognostic utility for short-term risk stratification (211).

The sFlt-1/PlGF ratio robustly captures angiogenic imbalance and rises before clinical onset, providing a mechanistic link between early placental dysfunction and later disease; however, it is most clinically useful later in gestation than the peri-implantation window (212).

First-trimester uterine artery pulsatility index (UtA-PI) offers a biophysical window on early uteroplacental perfusion and has been shown to predict subsequent PE (213). A 2024 meta-analysis concluded that first-trimester uterine artery pulsatility index (UtA-PI) is a useful predictor of PE and recommended its implementation within risk-stratification strategies, reinforcing the utility of early biophysical measures in identifying at-risk pregnancies (214). UtA-PI performs best when measured at standardized gestational ages and when combined with maternal factors in multivariable risk models (4).

Markers of decidual function and implantation quality, most notably IGFBP-1, map directly to endometrial decidualization and are biologically plausible indicators of implantation competence (215).

Single-cell and transcriptomic studies localize IGFBP-1–expressing stromal populations to the window of implantation and show altered decidual signatures in pregnancies complicated by placental disorders (216). Multimarker strategies that integrate biochemical (PAPP-A, PlGF), biophysical (UtA-PI) and clinical factors consistently outperform single markers for early-onset PE prediction in validation studies and clinical algorithms (4, 210). Important limitations temper clinical translation: effect sizes for individual early markers are modest, assay thresholds and sampling windows are heterogeneous, and much evidence originates from high-income populations with limited external validation (217). Prospective preconception and very-early pregnancy cohorts with standardized sampling, harmonized assays and adjudicated outcomes are required to test whether these early biomarkers reflect causal implantation pathology and to establish utility for targeted preventive trials. Table 2 *Early biochemical & biophysical biomarkers for PE (peri-implantation* → *1st trimester)* summarizes the earliest biochemical and biophysical markers with translational potential for implantation-centered risk stratification (sampling windows, evidence base and readiness).

**Table 2:** Early biochemical & biophysical biomarkers for PE (peri-implantation → 1st trimester).

Marker (type)	Biological rationale	Typical sampling window	Evidence/performance (summary)	Clinical readiness	Next steps/use in manuscript	Key citation(s)
PAPP-A (biochemical)	Reflects trophoblast/placental function and early placental growth; linked to decidual/trophoblast interactions.	8–13 weeks (first trimester).	Meta-analyses show lower first-trimester PAPP-A in pregnancies that develop PE; larger deficits for early-onset PE (e.g., MD ≈ −0.24 normalized units in pooled analyses). Alone modest discrimination; useful in panels.	Emerging → Research/Clinical (with panel)	List under “biochemical markers”; report pooled MD (cite meta) and note strongest signal for early-onset PE; recommend standardizing units/assays.	(209)
PlGF (biochemical)	Placental angiogenic factor produced early; low levels indicate impaired placental angiogenesis.	11–13 weeks (first trimester)—also informative later.	Consistently lower in pregnancies destined for PE; improves prediction when combined with PAPP-A and maternal factors (particularly for early/preterm PE).	Emerging → Research/Clinical (with panel)	Add as complementary marker to PAPP-A; recommend serial sampling studies to map peri-implantation trajectories.	(210)
sFlt-1/PlGF ratio (biochemical; angiogenic)	Reflects angiogenic imbalance; mechanistic link to placental dysfunction and maternal endothelial disease.	Best measured mid → late pregnancy for clinical use; trajectories informative earlier.	Robust predictor of short-term risk in symptomatic women; trajectory rises before clinical onset—mechanistic bridge from early placental dysfunction to overt PE. Less useful as stand-alone peri-implantation marker.	Validated (mid/late gestation)	Use in manuscript to link early implantation defects with later angiogenic imbalance; caution about timing (not primary early marker).	(212)
Uterine artery PI (UtA-PI) (biophysical)	Higher PI indicates impaired early uteroplacental perfusion/abnormal remodeling.	11–13 weeks (first trimester); measurement timing must be standardized.	Meta-analyses show UtA-PI predicts PE; combining UtA-PI with maternal factors improves detection of early-onset PE.	Emerging → Research/Clinical (in risk models)	Place under biophysical markers; recommend harmonized Doppler protocol and inclusion in multimarker risk models.	(213)
IGFBP-1 (decidual/biochemical)	Principal decidual product; marker of decidualization and implantation quality.	Peri-implantation → very early first trimester (preconception to ∼6–8 weeks depending on sampling).	Tissue and circulating studies show altered IGFBP-1 expression with impaired decidualization and adverse placentation; single-cell data localize IGFBP-1 + stromal cells to implantation window.	Research/Mechanistic	Use to link decidual competence to implantation-centered hypothesis; recommend targeted preconception/very-early pregnancy sampling studies.	(215, 218)
Endometrial/decidual transcriptomic signatures (molecular)	Capture cell-type specific decidual changes (immune, stromal, vascular) at implantation.	Preconception/window of implantation (cycle day ∼19–23) or early pregnancy biopsies (research only).	Single-cell and bulk transcriptomic studies identify altered decidualization signatures associated with later placental disease. Data are mechanistic and hypothesis-generating.	Exploratory/Research	Place in mechanisms subsection; recommend minimally invasive sampling and harmonized bioinformatics pipelines for multi-cohort validation.	(216)
Oxidative stress markers (placental/serum) (biochemical)	Early oxidative stress implicated in trophoblast dysfunction & impaired implantation.	Very early first trimester → ongoing.	Heterogeneous assays; several small studies show higher oxidative stress markers in pregnancies that later develop PE but standardization is lacking.	Exploratory/Research	Recommend assay harmonization and prospective validation; list as mechanistic candidate rather than validated predictor.	(219, 220)
Seminal-plasma cytokine/antigen exposure markers (exposure/biochemical)	Seminal antigens may prime maternal immune tolerance; cytokine milieu may modulate decidual immune adaptation.	Preconception (exposure history) or seminal fluid sampling pre-conception (research)	Observational studies associate greater cumulative seminal exposure with lower PE risk; cytokine marker data are preliminary.	Exploratory/Research/Translational	Include in epidemiology/immune priming section; propose partner-couple cohort studies and pilot interventional designs.	(114)
Composite multimarker algorithms (combined)	Combine biochemical+biophysical+maternal factors to optimize early prediction.	First trimester (commonly 11–13 weeks)	Validated algorithms (research/commercial) outperform single markers; sensitivity and specificity vary by population and implementation.	Promising → Research/Implementation with validation	Place as primary recommended strategy for early risk stratification; recommend external validation in diverse cohorts prior to clinical rollout.	(221, 222)

PAPP-A, pregnancy-associated plasma protein A; PlGF, placental growth factor; sFlt-1, soluble fms-like tyrosine kinase-1; UtA-PI, uterine artery pulsatility index; IGFBP-1, insulin-like growth factor binding protein-1. Clinical readiness: Validated, widely accepted for clinical use; Emerging, promising with substantial supporting evidence but requiring further validation; Exploratory, mechanistic/early research stage. Key sources: Tzanaki et al. (PAPP-A meta), PlGF systematic reviews, UtA-PI meta-analyses, sFlt-1/PlGF reviews.

To complement the biomarker summary in Table 2 and to align these markers with the implantation-centered framework proposed in this review, Table 3 maps each early biomarker to the specific implantation-era mechanism it reflects.

**Table 3:** Mapping early biomarkers to implantation-era pathophysiological mechanisms.

Biomarker	Implantation-era mechanism represented	Explanation (one sentence)
PAPP-A	Early trophoblast invasion; decidual–trophoblast interface	Low levels reflect impaired trophoblast growth and attachment.
PlGF	Early placental angiogenesis	Reduced early PlGF reflects insufficient early vascular development.
UtA-PI	Spiral artery remodeling/uteroplacental perfusion	High PI indicates poor remodeling and high-resistance early flow.
IGFBP-1	Decidualization competence	Key stromal product; early deficiency reflects suboptimal decidual setup.
Oxidative stress markers	Trophoblast microenvironment	Early oxidative imbalance impairs invasion and villous development.
Seminal-plasma markers	Immune priming before implantation	Seminal antigens modulate tolerance that affects implantation success.
hCG/hyperglycosylated hCG	Early trophoblast proliferation/differentiation	Altered trajectories indicate dysregulated transition to placentation.

### 14.1 Public health implications

Reframing PE as a disorder initiated during the implantation window has major public health implications. Early interventions targeting maternal immune adaptation, uterine receptivity, and peri-conceptional care could significantly reduce the incidence of PE and its associated maternal and neonatal complications.

Public health strategies should emphasize optimizing preconception health, promoting vaccination and infection prevention, minimizing exposure to environmental toxins, and enhancing reproductive health education. Policymakers must prioritize research and resource allocation toward interventions that target the critical peri-implantation period to improve maternal and perinatal outcomes globally.

### 14.2 Overlap with recent reviews and what this review adds

Several high-quality recent reviews have synthesized the evidence linking early maternal (decidual) dysfunction to later PE, and deserve explicit acknowledgement. A comprehensive disease primer summarized contemporary thinking on PE's multifactorial nature, including placental and maternal contributions (18). Tamara Garrido-Gómez and colleagues have repeatedly described the concept of decidualization resistance and provided key molecular and histologic evidence supporting a maternal-endometrium contribution to severe PE (19, 223). Recent debate over programmed frozen-embryo transfer (FET) and corpus-luteum absence highlights the growing epidemiologic concern that ART scheduling choices may modulate hypertensive risk (224). Finally, emerging multi-omic studies have begun to map decidualization resistance at cellular and molecular resolution, while reviews of seminal-plasma biology have reiterated its immunomodulatory role in endometrial receptivity (20).

Where our narrative overlaps with these works is in affirming that impaired decidualization and early maternal immune-endometrial interactions are important contributors to some forms of PE. However, this manuscript departs from prior reviews in three concrete ways. First, rather than restating that a maternal contribution exists, we explicitly integrate ART/programmed-FET epidemiology (and the corpus-luteum hypothesis) with peri-conception exposure biology (including seminal-plasma immune priming) to propose mechanistic linkages between exposures and implantation biology (95, 224). Second, we synthesize the latest multi-omic signatures of decidualization resistance and align them with specific immune-receptor (e.g., KIR–HLA) and CD40–CD40L pathway hypotheses to nominate testable peri-implantation biomarkers (17, 66, 198). Third, we translate these converging lines into an explicit, prioritized translational agenda—concrete cohort designs, biomarker sampling windows, and experimental approaches—aimed at moving from association to causal validation. This explicit mapping of exposures → implantation biology → biomarkers → study design is the principal novel contribution of this review.

## 15 Conclusion and future perspectives

PE is increasingly recognized as a disorder rooted not solely in late pregnancy placental dysfunction, but rather in early disruptions during implantation. This review highlights that impaired immune tolerance, oxidative stress, infections, genetic incompatibilities, and environmental insults during the critical implantation window collectively compromise trophoblast invasion and vascular remodeling, establishing the pathological foundation for PE.

By repositioning the pathogenesis of PE to the earliest stages of pregnancy, a new paradigm emerges, one that emphasizes prevention and intervention during preconception and peri-implantation periods rather than after clinical symptoms appear. Seminal plasma exposure, maternal immune priming, and uterine receptivity must be considered integral to early screening and therapeutic strategies. While we propose impaired implantation as a central trigger, it is likely one of several pathogenic pathways that converge on the common clinical phenotype of PE, and its contribution may vary across different patient subgroups.

Future research should prioritize the identification of biomarkers predicting implantation quality, the development of interventions enhancing maternal immune tolerance, and the refinement of assisted reproductive technologies to optimize the uterine environment. Clinically, there is an urgent need to recognize patients at risk based on implantation dynamics and to implement early preventive measures such as immunomodulation, antioxidant therapies, or lifestyle modifications prior to placental establishment. Ultimately, reframing PE as a disorder of impaired implantation provides opportunities to significantly reduce the global burden of maternal and fetal morbidity by targeting the origins of the disease at its earliest and most modifiable point.
